# Polymer Composite and Nanocomposite Dielectric Materials for Pulse Power Energy Storage †

**DOI:** 10.3390/ma2041697

**Published:** 2009-10-29

**Authors:** Peter Barber, Shiva Balasubramanian, Yogesh Anguchamy, Shushan Gong, Arief Wibowo, Hongsheng Gao, Harry J. Ploehn, Hans-Conrad zur Loye

**Affiliations:** Department of Chemistry and Biochemistry, Department of Chemical Engineering, University of South Carolina, Columbia, SC 29208, USA; E-Mails: barber@mail.chem.sc.edu (P.B.); wibowo@mail.chem.sc.edu (A.W.); Gong@mail.chem.sc.edu (S.G.); BALASUBS@cec.sc.edu (S.B.); ANGUCHAM@cec.sc.edu (Y.A.); GAO25@cec.sc.edu (H.G.)

**Keywords:** polymer, composites, dielectric, nanocomposite, dielectric breakdown strength, energy density, surface modification, barium titanate, titania, calcium copper titanate, lanthanum strontium nickelate

## Abstract

This review summarizes the current state of polymer composites used as dielectric materials for energy storage. The particular focus is on materials: polymers serving as the matrix, inorganic fillers used to increase the effective dielectric constant, and various recent investigations of functionalization of metal oxide fillers to improve compatibility with polymers. We review the recent literature focused on the dielectric characterization of composites, specifically the measurement of dielectric permittivity and breakdown field strength. Special attention is given to the analysis of the energy density of polymer composite materials and how the functionalization of the inorganic filler affects the energy density of polymer composite dielectric materials.

## 1. Introduction

Electrical energy storage plays a key role in mobile electronic devices, stationary power systems, hybrid electric vehicles, and pulse power applications [1,2]. In particular, there is a growing need for capacitors that can accumulate a large amount of energy and then deliver it nearly instantaneously. This kind of “pulse power” is needed for a variety of military and commercial applications. Over time, these applications demand ever higher energy and power densities as well as higher rate capability.

Dielectric materials can be used to store electrical energy in the form of charge separation when the electron distributions around constituent atoms or molecules are polarized by an external electric field. The complex permittivity of a material can be expressed as:

(1) $$\varepsilon^{*}=\varepsilon^{'}-j\varepsilon^{"}$$

where *ε*^'^ and *ε*^ʺ^ are the real and imaginary parts of the complex permittivity and j = √-1. The magnitudes of *ε*^'^ and *ε*^ʺ^ depend on the frequency *ω* of the applied electric field and are related by the Kramers-Kronig relation:

(2) $$\varepsilon^{ʹ}(\omega )=\varepsilon_{o}+\frac{2}{\pi }\int_{0}^{\infty }\frac{u\varepsilon^{\prime \prime }(u)}{u^{2}-\omega^{2}}du$$

The real part of the permittivity is given by:

(3) $$\varepsilon^{'}=\varepsilon_{o}\varepsilon_{r}$$

where ε_o_ is the permittivity of vacuum (8.85 × 10^-12^ F/m) and ε_r_ is the relative permittivity or dielectric constant of the material. The magnitude of *ε*^'^ (or the dielectric constant ε_r_) indicates the ability of the material to store energy from the applied electric field. A parallel plate capacitor with area *A* and thickness *d* has a capacitance given by:

(4) $$C=\varepsilon_{o}\varepsilon_{r}A/d$$

The imaginary part of the permittivity, *ε*^ʺ^, is called the dielectric loss. As the polarization of a material under an applied electric field varies, some of the field energy is dissipated due to charge migration (i.e., conduction) or conversion into thermal energy (e.g., molecular vibration). For an energy storage device like a capacitor, we obviously wish to minimize the dielectric loss. 

Ceramic capacitors based on highly polarizable inorganic materials have traditionally been used to meet the need for pulse power applications. The energy stored by a capacitor is given by:

(5) $$W=\frac{1}{2}CV_{bd}^{2}$$

where *V_bd_* is the breakdown voltage. In terms of the dielectric constant (ε_r_), the volumetric energy density of a dielectric capacitor is:

(6) $$\overset{˘}{W}\equiv \frac{W}{Ad}=\frac{1}{2}\varepsilon_{0}\varepsilon_{r}E_{bd}^{2}$$

where *E_bd_* ≡ *V_bd_*/*d* is the breakdown field strength. Despite having high dielectric constants (Table 1), ceramic capacitors have low inherent breakdown field strength, which results in low energy density. Moreover, it is difficult to manufacture ceramic capacitors with the desired high capacity for energy storage.

**Table 1:** Dielectric permittivity values of commonly used ceramics for capacitors [1]. PMN-PT (65/35) is the abbreviation for 65 % Lead magnesium niobate and 35 % Lead titanate. PLZT (7/60/40) is the abbreviation for Lead lanthanum zirconium titanate.

Composition	Dielectric permittivity
BaTiO_3_	1,700
PMN-PT (65/35)	3,640
PbNb_2_O_6_	225
PLZT (7/60/40)	2,590
SiO_2_	3.9
Al_2_O_3_	9
Ta_2_O_5_	22
TiO_2_	80
SrTiO_3_	2,000
ZrO_2_	25
HfO_2_	25
HfSiO_4_	11
La_2_O_3_	30
Y_2_O_3_	15
α-LaAlO_3_	30
CaCu_3_Ti_4_O_12_	~60,000
La_1.8_Sr_0.2_NiO_4_	~100,000

Polymers, on the other hand, are easily processed into large area films, and several polymers have relatively high breakdown field strengths. Unfortunately, they also typically have low dielectric constants (Table 2), and thus low energy densities.

**Table 2:** List of dielectric permittivities of commonly used polymers in capacitors [1].

Polymer	Dielectric permittivity
Nonfluorinated aromatic polyimides	3.2-3.6
Fluorinated polyimide	2.6-2.8
Poly(phenyl quinoxaline)	2.8
Poly(arylene ether oxazole)	2.6-2.8
Poly(arylene ether)	2.9
Polyquinoline	2.8
Silsesquioxane	2.8-3.0
Poly(norborene)	2.4
Perfluorocyclobutane polyether	2.4
Fluorinated poly(arylene ether)	2.7
Polynaphthalene	2.2
Poly(tetrafluoroethylene)	1.9
Polystyrene	2.6
Poly(vinylidene fluoride-co-hexafluoropropylene)	~12
Poly(ether ketone ketone)	~3.5

### 1.1. Dielectric Permittivity

#### 1.1.1. Polymer Composite Dielectrics

The need for pulse power energy storage systems with high energy density has led to the development of polymer composite systems that combine the processability and breakdown field strength of the polymer with the high dielectric constant of ceramic fillers. Ideally, the fillers help to increase the effective dielectric constant of the composite system without compromising the high inherent breakdown strength of polymers. Moreover, increasing the effective dielectric constant must be achieved without an unacceptably large increase in dielectric loss (i.e., energy dissipation). In reality, the objectives of high dielectric constant, high breakdown field strength, and low dielectric loss are not likely to all be achieved; the best solution will be a compromise. Consequently, much research is being carried out to develop improved polymer composite materials through a better understanding of the physical phenomena governing composite dielectric permittivity and breakdown field strength. As both of these issues are likely to involve the polymer-filler interface, research seeking a better understanding the chemistry and structure of the filler-polymer interface is a priority.

Most of the current studies on dielectric polymer composites focus on the enhancement of the dielectric permittivity using ferroelectric metal oxides Pb(Zr,Ti)O_3_ (PZT), Pb(Mg_0.33_Nb_0.77_)O_3_-PbTiO_3_ (PMNT), and BaTiO_3_ (BT). From the point of view of increasing the composite’s effective dielectric constant, the availability of inorganic fillers with dielectric constants on the order of hundreds and even thousands makes it very appealing to introduce them into polymers, which generally possess dielectric constants less than 10. However, the resulting composites’ effective dielectric constants generally fall short of expectations. Specifically, since the filler has a much greater permittivity than that of the polymer matrix, most of the increase in the effective dielectric constant comes through an increase in the average field in the polymer matrix, with very little of the energy being stored in the high permittivity phase. Also, the large contrast in permittivity between two phases can give rise to highly inhomogeneous electric fields. Lastly, incompatibility between the organophilic polymer matrix and the hydrophilic metal oxide filler impedes the formation of a homogenous composite. Thus, a major research direction in this field remains focused on modifying the inorganic filler surface to compatibilize the inorganic filler with the polymer matrix. Highly inhomogeneous fields and structural inhomogeneity generally lead to a significant reduction in the effective breakdown field strength of the composite, limiting the increase in the energy storage capacity and energy density. Clearly there are several persistent issues that need to be overcome to increase the energy density and capacity of dielectric polymer composite materials.

#### 1.1.2. Models for Effective Dielectric Constant

Many theoretical approaches have been developed to predict the effective dielectric constants of polymer composite systems. The volume-fraction average is a simple (but inaccurate) method to estimate the effective dielectric constant of a polymer composite material:

(7) $$\varepsilon_{eff}=\phi_{1}\varepsilon_{1}+\phi_{2}\varepsilon_{2}$$

where the subscripts 1 and 2 represent the polymer and the ceramic phase, respectively, and *ϕ* is the volume fraction of the constituents. According to the volume-fraction average model (Equation 7), the effective dielectric constant of the composite increases sharply at low volume fraction of the ceramic filler. Many studies involving both experiments [3,4] and theory [5,6] disprove the trend predicted by Equation 7. 

More realistic models are based on mean field theory. The Maxwell equation:

(8) $$\varepsilon_{eff}=\varepsilon_{1}\frac{\varepsilon_{2}+2\varepsilon_{1}-2(1-\phi_{1})(\varepsilon_{1}-\varepsilon_{2})}{\varepsilon_{2}+2\varepsilon_{1}+(1-\phi_{1})(\varepsilon_{1}-\varepsilon_{2})}$$

is based on a mean field approximation of a single spherical inclusion surrounded by a continuous matrix of the polymer [7]. Thus Maxwell’s equation is strictly valid only as the filler fraction goes to zero, i.e., infinite dilution.

Another mean field theory, known as the Bruggeman model, treats the binary mixture as being composed of repeated units cells composed of the matrix phase with spherical inclusions in the center [7]. The effective dielectric constant (*ε_eff_*) of the binary mixture is given by:

(9) $$\phi_{1}\left(\frac{\varepsilon_{1}-\varepsilon_{eff}}{\varepsilon_{1}+2\varepsilon_{eff}}\right)+\phi_{2}\left(\frac{\varepsilon_{2}-\varepsilon_{eff}}{\varepsilon_{2}+2\varepsilon_{eff}}\right)=0$$

**Figure 1:**
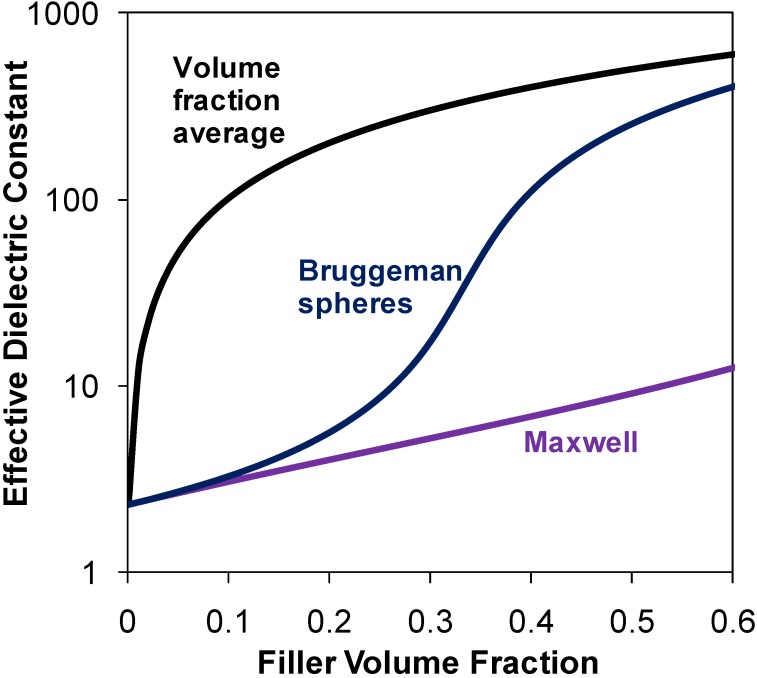
The dielectric constant predicted by various models for a blend of inorganic spheres (ε_2_ = 1,000) dispersed in a polymer matrix (ε_1_ = 2.3).

Figure 1 shows the dielectric constant predicted by various models for a blend of inorganic spheres (ε_2_ = 1,000) dispersed in a polymer matrix (ε_1_ = 2.3). Various studies have indicated that the effective dielectric constant predicted by the Bruggeman equation increases sharply for filler volume fractions above 20% and can be very high for ceramic particle loadings higher than 50% by volume. 

In two-phase models, both the constituents of the composite system are considered as different phases rather than considering one constituent of the composite as an inclusion in a continuous phase of another. Rao *et al*. made numerical predictions of the effective dielectric constant for polymer-ceramic composites based on effective-medium theory (EMT) [8]. They proposed that when the particle size of the ceramic filler is small compared to that of the composite, the dielectric permittivity of the composite could be calculated in terms of an effective medium whose dielectric permittivity can be obtained by averaging over the dielectric permittivity of the two constituents. They introduced an arbitrary fitting parameter in their model to account for the irregular morphology of the ceramic particles. The EMT model equation fit the experimental results for lead magnesium niobate-lead titanate/epoxy composites with less than 10% error. However, the values of the arbitrary fitting parameter in this model are difficult to rationalize.

Much theoretical work has been carried out to account for the role of the interface between the polymer phase and the ceramic phase because the interface plays an important role in determining the performance of the dielectric materials. These models treat the “interphase” as a separate phase in addition to the filler and polymer phases.

Todd *et al*. developed a model called the “interphase power law” to study the complex permittivity of the composite system [9]. The model takes into account the permittivity and the volume fractions of the polymer, filler and the interface region. The effective permittivity determined by the model was compared with experimental results. The model provides insight into the contributions of the interface towards the dielectric permittivity and suggests the interface as the reason for deviations of the predicted permittivity from the predictions of the standard mixture models. 

The model developed by Vo *et al*. considered the contribution of molecular polarizability at the interface to the effective dielectric constant [10]. They based their model on the dielectric constant ratio between the filler and the polymer matrix and the degree of interaction between the filler and the matrix. They introduced a parameter, termed the interface volume constant, which accounted for the matrix/filler interaction strength. A value of zero signifies negligible interaction between the matrix and the filler, while large positive values indicate strong interactions. They also suggested that the interface volume constant should depend on the size of the filler.

Later Murugaraj *et al*. applied the Vo-Shi model to rationalize the high dielectric constant values obtained for polyimide-alumina nanocomposite thin films [11]. The measured dielectric constant values were well above those predicted by Maxwell’s equation (Equation 8). The values predicted by the Vo-Shi model were in agreement with the experimental values after fitting the data using large positive values for the interface volume constant, implying a strong interaction between the polymer and the filler particles. They also suggested the formation of interfacial dipoles with high molecular polarizability for achieving enhanced dielectric performance. 

Along with mathematical modeling, numerical simulations have been carried out to compute the effective dielectric constant. Tuncer *et al*. performed numerical calculations using the finite element method to investigate the frequency dependent dielectric properties of binary dielectric mixtures. They found that the dielectric characteristics exhibited by the mixtures were due to interfacial polarization. Moreover, they reported that the dielectric relaxation was strongly dependent on the conductivity of the phases and the topology of the dielectric mixtures [12,13]. Myroshnychenko *et al*. used the finite-element modeling method for prediction of the complex effective permittivity of two-phase random statistically isotropic heterostructures [4]. They assumed the composite to be a distribution of one dielectric phase randomly dispersed in a continuous matrix of another dielectric phase. The numerical values for the effective complex permittivity were found to lie between the curves corresponding to the Maxwell and Bruggeman equations. 

#### 1.1.3. Nanocomposite Dielectric Concepts

Lewis proposed that as the size of filler particles decreases to the nanometer scale, the properties of the polymer-filler interface would become dominant over the bulk properties of the constituents [14,15]. This concept goes beyond that of modifying the filler surface to achieve better dispersion of the dielectric filler particles. In a true nanocomposite dielectric or “nano-dielectric”, the unique properties of the interface are amplified by the high surface area of the filler.

Experimentally, this concept was explored by Sun *et al*. who studied the influence of the interface on the dielectric properties of epoxy/silica composites [16]. The dielectric properties of both micron-sized and nano-sized silica were tested. The dielectric permittivity and the loss factor were found to be higher for nanocomposites than for micro-composites at low frequencies. The higher dielectric loss for the nanocomposites was attributed to the enhanced ionic conductivity caused by the contaminants from the sol gel synthesized nano-sized silica. 

Theoretical models have also been extended to consider the influence of interface on nano-dielectrics. Tanaka *et al*. proposed a multi-core model to understand the dielectric properties of polymer nanocomposites [17]. They proposed that the interface of a spherical inorganic filler particle embedded in polymer matrix consists of three distinct regions: a bonded layer (first layer), a bound layer (second layer) and a loose layer (third layer), with an electric double layer overlapping the three layers. The first layer corresponds to a contact layer where the polymer is in intimate contact with the filler surface, and the second layer corresponds to the interfacial region. Finally, there is the third layer, where the properties of the bulk polymer are approached. The second layer contributes to the reduction in the permittivity by disturbing the motion of dipoles originating from some polar groups. The free volume in the composite, mostly associated with the third layer, also causes a reduction in the dielectric constant of the composite. Though the first layer directly links the particle to the polymer and establishes direct contact, the second and the third layers are suggested to be most influential in affecting the dielectric properties of the polymer composite. By affixing suitable organic groups to the filler particle, one can directly impact the interfacial region. The selection of the surface organic groups, in terms of polarity, polarizability, mobility, and size could have a major impact on the dielectric properties of the polymer nanocomposites.

Tuncer *et al*. evaluated the dielectric properties of cobalt iron-oxide (CoFe_2_O_4_) nanoparticle modified poly (methyl methacrylate) nanodielectric system [18]. With the addition of small quantites of CoFe_2_O_4_, the dielectric permittivity of the nanocomposite decreased. Numerical modeling was performed to analyze the dielectric data. They found that the relaxation behavior of the composite system at high frequencies was different from that of the unfilled polymer. This phenomenon was suggested as arising from interfacial polarization. Also, the breakdown data analyzed using the two-parameter Weibull expression revealed an improvement in the dielectric breakdown characteristics with the addition of the nanoparticle.

### 1.2. Dielectric Breakdown

#### 1.2.1. Breakdown Behavior of Polymers

Dielectric breakdown is the catastrophic failure of an insulating material under an external applied field resulting in mechanical damage and electrical conduction, depending upon the defect density of the solid material. Electrical breakdown testing of polymers for insulator applications has long been a subject of interest. Table 3 is a compilation of the dielectric strengths of some common polymers [19]. 

**Table 3:** List of dielectric strengths of insulating polymers [19].

Polymer	Dielectric Strength (V/μm)
Polyethylene (LD)	200
Polyethylene (HD)	200
Polyethylene (XL)	220
Polypropylene (Biaxially oriented)	200
Polystyrene	200
Polytetrafluoroethylene	88-176
Poly(vinylidene fluoride)	10.2
Polycarbonate	252
Polyester	300
Polyimide	280
Epoxy resin	25-45

In order to improve insulator performance, it is essential to understand the mechanisms of electrical breakdown in solid dielectrics. However, unlike the case for gases, electrical breakdown and conduction mechanisms in polymeric solids are less understood. In solid dielectrics, the electrical transport phenomena include currents due to orientation and interfacial polarization in addition to electronic and ionic charge carriers. Vorob’ev proposed that the phenomenon of electrical breakdown in solid dielectrics is a solid-to-plasma phase transition with energy gained from external field [20]. 

The most common modes of electrical breakdown for solids are thought to be intrinsic, thermal and ionization mechanisms [21,22]. Intrinsic electric strength represents the “true” strength of a material and depends only on the material and the temperature. An insulator material is assumed to have reached its intrinsic strength when the applied field is sufficient to raise electrons from the valence band to the conduction band. It is achieved under carefully controlled test conditions designed to create a high stress at the center of test specimen to eliminate external discharges. Thermal breakdown represents failure due to localized, non-uniform fields due to conduction currents and dielectric losses arising from polarization under an electric field. This occurs at a critical current density, which can be avoided by controlling the experimental conditions. 

Another mechanism is the avalanche mechanism, which is thought to be one of the most common mechanisms of dielectric breakdown in polymers. Avalanche or ionization breakdown occurs when free electrons in polymers acquire high energy by accelerating along the mean free path to knock out other electrons, leading to conduction due to large multiplication of electrons in the conduction band. The amorphous phase in polymers with unoccupied volume consisting of holes of molecular order presents a free path for the electrons to accelerate under an external field and gain energy. This is the basic idea of Free Volume Theory proposed by Artbauer [23]. Sabuni and Nelson studied the effect of plasticizers on electric strength of Polystyrene at different temperatures [24]. They found that increasing the temperature led to the reduction in the dielectric strength with a sharp reduction at a critical temperature, which was indistinguishable from the glass transition temperature. The presence of a plasticizing agent strongly influenced the dielectric strength and the critical temperature. They attribute the breakdown to higher mobility of free electrons leading to electron avalanches at higher temperatures. Wiacek has investigated the addition of cyclo-aliphatic hydrocarbon units to fluorinated polyesters [25]. These hydrocarbons have high order and are similar to diamond in their molecular structure and electrical properties. Wiacek has observed that poly 9,9-bis(4-hydroxyphenyl) fluorine end-capped with 4,9-diamantane had the highest breakdown strength resulting in a high energy density. Furthermore, the addition of a cross-linking agent, *Cyclotene,* and subsequent curing increased the breakdown strength by 16%.

Various physical modifications of polymers have been made in an attempt to improve the materials’ dielectric breakdown strength. Hosier *et al*. investigated blends of linear and branched polyethylene (PE) and found that PE blends showed a higher breakdown strength compared to linear PE. [26] They cite the network of thick lamellae structure that changed local charge transport processes resulting in high breakdown strength for blends. Schneuwly *et al*. successfully increased the breakdown strength of polypropylene (PP) films by more than 25% by impregnation with rape-seed oil [27]. They argued that the oil filled the voids of the amorphous region of the polymer and increased the overall dielectric strength. However, they observed a decrease in the breakdown strength of PP with increasing temperature when small quantities of low molecular weight organic and inorganic additives were incorporated into the polymer. They argued that as temperature increased, the amorphous regions softened, creating more free volume that resulted in lower breakdown strength. Alternatively, chemical modifications have also resulted in improved dielectric strength of polymers, as shown by Job *et al*. [28] They have increased the breakdown strength of poly(ethylene terephthalate) (PET) films by *in-situ* polymerization of a layer of polyaniline (PANI). A 30% increase in dielectric strength was observed when the non-conductive PANI filled the voids of PET. Breakdown was proposed to occur by the electron avalanche process.

Breakdown in polymers with saturated bonds, such as polyethylene and polypropylene, cannot be explained by the electron avalanche mechanism as they have a short free path length. As a result, the electrons cannot gain sufficient energy by acceleration to cause an avalanche. In these polymers, electrical breakdown is the last step in a process of polymer degradation that results in the formation of conducting channels. The formation of these conducting channels depends on the intensity and time of the applied external field. The lifetime of these polymers decreases exponentially with applied electric field [29].

Overall, a general consensus has emerged that the reduction in dielectric breakdown strength of polymers is closely related to the dissociation (bond scission) of polymer molecules under an applied external field. The dissociation process is considered to be the initiation of polymeric breakdown. However, the exact mechanism of dissociation is unclear. One approach to explain this phenomenon is the non-uniform distribution of electric field intensity within a polymer due to electrode defects and inherent differences within the structure of polymer [29]. The different degrees of ordering of macromolecules and the presence of regions of varying densities gives rise to different conductivities under an applied field. A non-uniform voltage drop occurs within the polymer when a current begins to flow, leading to variable distribution of electric field. A detailed analysis of breakdown processes in polymers has been discussed by Ieda *et al.* [30]. 

#### 1.2.2. Breakdown Behavior of Polymer Composites and Nanocomposites

Improving the dielectric properties of polymers is an active research field. Inorganic fillers have been added to polymers in an attempt to increase the effective dielectric constant and energy density [18]. In conventional composites, typical filler particles are micron-sized or larger. Unfortunately, the addition of micron-sized fillers has often had a negative impact on breakdown strength. [31] This may be due to aggregation of filler particles, which are thought to introduce defect centers that distort and enhance the local electric field, resulting in reduced breakdown strength. This field distortion is primarily due to the difference in permittivities of the filler and the polymer matrix under AC conditions, and mainly due to differences in conductivities under DC conditions. [32] Also, as the particle size increases, the probability of field enhancement increases. 

In order to overcome the limitations of conventional composites, the concept of polymer nanocomposites has been proposed in which the filler particles have nanometer dimensions [14,33]. Nanocomposites differ from microcomposites in three aspects - they contain small amounts of fillers, the filler particles have sizes in the range of nanometers, and the filler-polymer interfacial area is large. In the presence of a few weight percent of anisotropic fillers with high aspect ratio, the bulk polymer is converted into an interfacial polymer with (hopefully) favorable properties. In microcomposites, the composite properties are typically a weighted average of the constituent material properties. This is not the case in nanocomposites due to the dominant role of the interface. Tanaka *et al*. have identified a large number of nanocomposite systems for a variety of applications [34].

The interface plays a critical role in defining the properties of nanocomposites. As a result of nanofiller addition, a reduction in the internal field due to the decrease in particle size is observed [35]. Since large particle size leads to greater field distortion resulting in field enhancement, a nanocomposite should show a lower breakdown loss compared to a microcomposite. Also, changes in the space charge distribution have been proposed as a reason for improvements in the dielectric behavior of the nanocomposites [36]. The introduction of a nano-filler significantly reduces charge accumulation, leading to a reduced probability of breakdown at lower fields. 

Tanaka *et al*. have proposed a multi-core model that describes charge behavior and interfacial structure [17]. The interface is thought to consist of bonded, bound and a loose layer. The bonded layer arises when polymer is tightly bonded to the particle, as often observed in case of nanocomposites. The bound layer that overlaps the bonded layer corresponds to a strong bond between the polymer chains and the first layer. The third layer is a “loosely coupling interaction” layer that contains free volume and the crystalline region of the polymer matrix. Based on this multi-core model, Smith *et al*. proposed a mechanistic hypothesis of the interface structure [37]. Due to the presence of different layers, a gradient in the charge mobility is established. The differences in the Fermi levels between the nanoparticles and the polymer gives rise to a surface charge on the nanoparticle. In order to maintain charge neutrality at the interface, a redistribution of charge occurs at the interface leading to a Helmholtz or Stern layer. A diffuse double layer of charge in the matrix polymer exists far away from the interface. 

In order to understand how the above description of interfacial structure has a positive effect on the dielectric properties of nanocomposites, the following idea has been proposed. The diffuse double layer in the polymer is a region of higher charge mobility and strongly influences the dispersion and dielectric properties of the composite. This double layer, in turn, depends on the charge in the Stern layer. Suitable alteration of the interface results in changes in mobility, free volume and trap sites for charge carriers (in this case, electrons). This could explain the higher breakdown strength observed in nanocomposites developed by Ma *et al*. They modified titanium dioxide with a polar silane coupling agent and observed a decrease in the mobility of charge carriers in their nanocomposites [35]. The presence of filler in low concentrations eliminates overlapping of local conductive regions and thus prevents premature global breakdown in specimens. 

Surface functionalization of nanofillers does not always improve the dispersion of fillers, as observed by Ma *et al.* in the same work. Although the introduction of polar silane modified TiO_2_ nanoparticles in a polyethylene matrix resulted in an agglomeration due to an incompatible interface, higher breakdown strength was observed in composites containing surface-modified TiO_2_ nanoparticles than in composites containing unmodified TiO_2_. The reason cited was improved electron scattering by the polar interfacial groups and a decrease in the degree of polymer crystallinity. In contrast, Kim *et al*. reduced the aggregation of BaTiO_3_ nanoparticles by modifying the particle surface with phosphonic acid groups [38]. They report an increase in dielectric constant and decrease in the breakdown strength of the nanocomposites compared to the base polymer. Similar observations were made by Li *et al*., who argued that the increased energy density of poly(vinylidene fluoride) nano-BaTiO_3_ composites is due to the enhanced electric displacement of nanoparticles and not due to changes in the crystallinity of the polymer [39]. Although the degree of crystallinity did not significantly affect the polymer’s breakdown strength in their work, they speculated that the crystalline phase can enhance charge transport due to the ordered structure.

Nanoparticles are believed to disrupt the continuity of the path provided to charge carriers resulting in higher breakdown strength. Roy *et al*. have found that the addition of untreated and surface-treated nanosilica to cross-linked polyethylene resulted in a lower dielectric constant compared to the base polymer [40]. This was not the case in micron-filled composites, which showed higher dielectric constants than predicted by various mixing rules. They attributed the higher dielectric constants in microcomposites to interfacial polarization, or the accumulation of charge in a local environment as they drift through the material. Although this increase in the dielectric constant is desirable, microcomposites showed much lower breakdown strengths compared to the base polymer. On the other hand, nanocomposites showed at least a 15% increase in breakdown strength compared to the pure polymer at room temperature. The authors argue that the increase in breakdown strength of nanocomposites may be attributed to the reduction in chain movement of the polymer through physical bonding or confinement, as suggested by the elimination of a broad loss peak. The advantage of adding nano-fillers is that they conform to the chain length of the polymer and hence reduce Maxwell-Wagner-Sillar type interfacial polarization arising from the differences in dielectric permittivity of the polymer and filler.

Clearly, the choice of surface groups affects the interfacial structure and is important for improving the dielectric properties of filled polymers. However, there is little agreement on how interfacial modification affects dielectric breakdown. 

#### 1.2.3. Statistical Analysis of Breakdown Data

Although a number of samples of the same material may be tested, each sample might exhibit breakdown at a different voltage due to random structural and measurement differences. The breakdown voltage (or time to breakdown in constant stress testing) can be considered as a random variable, which necessitates a statistical analysis of the breakdown data. Electrical breakdown can be considered as a system failure under the condition where a weak link in the system fails. The Weibull distribution, which is a type of extreme value distribution, is the most common for such applications, and has been well developed for the analysis of small and large data sets with censored data. Other distributions used for electrical breakdown are the Gumbel and Lognormal [41]. Alternatively, Tuncer *et al*. have proposed a different expression for breakdown analysis [42]. The Weibull probability density function in three parameters is given by:

(10) $$f(x)=\frac{\beta }{\alpha }{\left(\frac{x-\gamma }{\alpha }\right)}^{\beta -1}\;\text{exp}{\left(-\frac{x-\gamma }{\alpha }\right)}^{\beta }$$

where α (V/μm), known as the scale parameter, is the electric field at which at least 63.2% of the samples are bound to fail. The parameter β (dimensionless), known as the shape parameter, is a measure of scatter in the data. A high value of β corresponds to lower scatter. For polymers, β values in the range of 2-4 are commonly observed, as reported by Roy *et al*. [38]. However, very high values for β are also found in the literature [43]. The third parameter, denoted by γ (V / μm), is the location parameter and represents the minimum breakdown voltage of the specimen below which none of the samples will fail. This parameter should be less than half of the lowest breakdown voltage observed, but usually is set to zero as there is no convincing physical explanation for non-zero values. The Weibull probability density function indicates the probability of a Weibull random event as a function of the independent variable *x*. Assuming the dielectric breakdown is a random process that follows the Weibull distribution, *f(x)* is the probability of dielectric breakdown at an applied field *x*. The cumulative distribution function:

(11) $$F(x)=\int_{0}^{x}f(u)du=1-\text{exp}{\left(-\frac{x}{\alpha }\right)}^{\beta }$$

gives the overall probability that a sample undergoes dielectric breakdown under an applied electrical field less than or equal to *x*.

Benard’s approximation of median ranks [44] was used to rank samples in increasing order of their observed breakdown strength before plotting the failure probability as a function of breakdown strength values. It serves to determine the unreliability of each failure and gives a 50 % confidence level of the true failure being the *i^th^* failure among *n* samples. This approximation is given by:

(12) $$f(i,n)=\frac{i-0.3}{n+0.4}$$

## 2. Inorganic Additives for Polymer Composite Dielectrics

### 2.1. Barium Titanate (BaTiO_3_) Composites

#### 2.1.1. Barium Titanate (BaTiO_3_)

**Figure 2:**
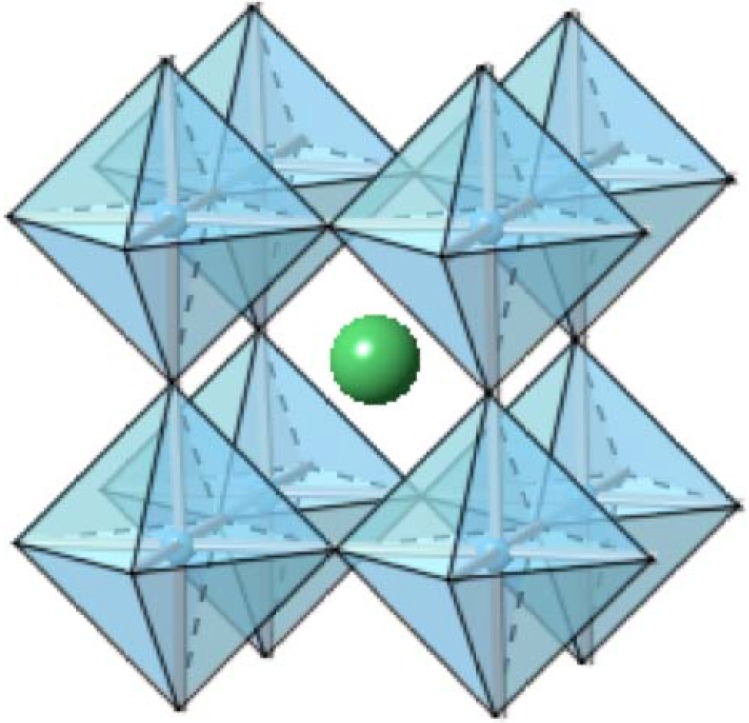
A cubic *ABO_3_* (BaTiO_3_) perovskite-type unit cell, with Ba^2+^ ions shown in green, and TiO_6_ corner-sharing octahedra in blue.

Perhaps the most widely investigated oxide in the field of dielectrics, barium titanate was first studied in the 1950s. Barium titanate (BT) belongs to a group of materials that crystallize with the perovskite structure, as shown Figure 2, and that have the general composition of *ABO_3_*. Many other ferroelectric ceramics, such as BT, lead titanate (PbTiO_3_), PZT, PLZT, PMN, and potassium niobate (KNbO_3_) also belong to the perovskite family. Barium titanate exists in the paraelectric cubic phase above its Curie point of about 130 °C, while in the temperature range of 0 °C to 130 °C, the ferroelectric tetragonal phase is stable. 

**Figure 3:**
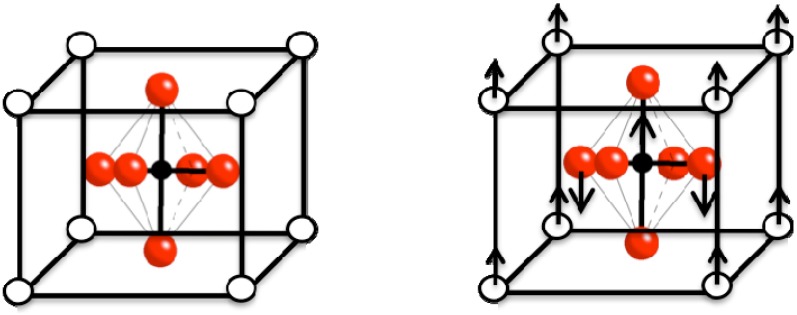
The crystal structure of BaTiO_3_ above the Curie point is cubic (left); below the Curie point the structure is tetragonal due to the displacement of the Ba^2+^(white) and Ti^4+^(black) ions relative to the O^2-^(red) ions as indicated by the arrows.

Barium titanate’s impressive dielectric properties arise from a structural change where the center Ba^2+^ and Ti^4+^ cations are displaced relative to the O^2-^ ions, leading to the formation of electric dipoles (Figure 3). This spontaneous polarization is the net dipole moment produced per unit volume for the dipoles pointing in a given direction. Various degrees of *A-* and *B-* site substitutions have been tried to study their effect on the dielectric and ferroelectric properties of BT. For example, increasing Sr^2+^ substitutions on the *A-*site has been found to reduce the Curie point linearly to room temperature, while the substitution of Pb^2+^ for Ba^2+^ raises the Curie point. The simultaneous substitution into both *A-* and *B-*sites with different cations has been used to tailor the properties of BT [40,45,46].

Interestingly the dielectric properties of BT have been found to be grain size dependent [47,48,49]. The room temperature dielectric constant of coarse grain (10 micron) BT ceramics is found to be in the range of 1,500–2,000, while fine grained (~1 micron) BT ceramics exhibit an enhanced room temperature dielectric constant of between 3,500–6,000. The grain size effect on the dielectric constant at room temperature has been explained by Buessem *et al*., who propose that the internal stresses in fine grained BT must be much greater than in the coarse grained ceramic, thus leading to a higher permittivity at room temperature [50]. However, further miniaturization of the particle size will lead to grain-sizes too small to sustain a sufficient dielectric constant and therefore reduce the bulk dielectric constant of the material. Grain size effects will be discussed again later with respect to the incorporation of high dielectric materials into polymer systems as well as with respect to the observation of ultra high dielectric constants in some materials.

Conventional high-permittivity materials, such as BT, can be processed into thin films by using chemical solution deposition followed by high temperature processing yielding a relative permittivity of about 2500 and a relatively low dielectric loss. However, the high-temperature sintering is not compatible with many substrate materials [51]. As mentioned previously, various approaches to achieve high dielectric permittivity materials based on nanocomposites containing nano-particles have been pursued. Such nanocomposites have afforded high dielectric permittivities, however, the resulting materials are limited by high-temperature processing requirements that can lead to high dielectric loss or low dielectric strength [52,53,54]. Simple solution processing of BT particles in a polymer host generally results in poor film quality and inhomogeneities that are mainly caused by agglomeration of the BT particles. Several approaches to prevent particle agglomeration have been reported in the literature. Specifically, the addition of surfactants, such as phosphate esters and oligomers, can improve the dispersion of BT particles in host polymers [8,55]. However, the addition of surfactants can also lead to an increase in the dielectric loss and the leakage current due to residual free surfactant [56]. Therefore, researchers have started to use chemical bonds to bind chemical modifiers to BT. Ramesh *et al*., for example, have reported on the use of trialkoxysilane surface modifiers to achieve dispersion of BT nanoparticles in epoxy polymer hosts [57], while Kim *et al*. performed a systematic study of identifying ligands that can form stable bonds to a BT surface through coordination or condensation reactions [38]. They concluded that phosphonic acid ligands effect robust surface modification of BT and, furthermore, generate well-dispersed BT-nanoparticles that afford high dielectric permittivity polymer-composite films.

Similarly, Wang *et al*. synthesized crystalline nanosize BT from the decomposition of ethylenediamine-modified titanium(IV) isopropoxide and barium hydroxide in aqueous solution. This affords ethylenediamine-capped BT, which showed a marked improvement in its ability to be dispersed in organic solvents [39]. The above modifications of BT are by no means a complete list of functionalization of BT. They instead represent the most recent advances in the field for BT functionalization, which is necessary to enhance the compatibility of BT with organic polymers. We will now discuss how these modifications impact the overall energy density of BT-polymer composites.

#### 2.1.2. BT Polymer Composites 

In general, the energy density of a biphasic-composite is the sum of the energy density of each constituent. In most of the ceramic-polymer composites, the volume fractions of the two constituents are of the same order of magnitude. Therefore, to achieve a high energy density of a composite, a significant contribution toward the total energy density from each constitute is needed. Polymers currently used as matrices in dielectric nanocomposites, including polyethylene, poly(methyl methacrylate), epoxy resins, and polyimides, usually possess dielectric permittivities of ~2-5, which are significantly lower than those found in their inorganic counterparts. This greatly limits the energy density obtained in the polymer matrix, and consequently, in the resulting composites.

**Figure 4:**
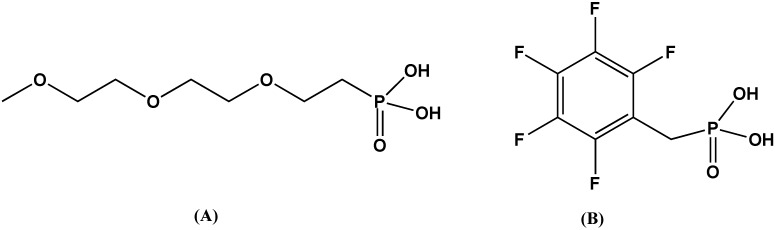
Structures of the phosphonic acid ligands used to modify BT for dispersion into polymers, where A) {2-[2-(2-methoxyethoxy)ethoxyethyl}phosphonic acid, and B) pentafluorobenzyl phosphonic acid.

As mentioned earlier, Kim’s group determined that phosphonic acids bind very well to the surface of BT. Having demonstrated the effective binding of phosphonic acids to BT, they examined the use of phosphonic acids bearing specific functionalities to control the interfacial interactions between BT particles and the dielectric host materials and, thus, facilitate the formation of homogeneously dispersed, high dielectric permittivity nanocomposites. Kim chose two types of polymer host materials: a low permittivity bisphenol A-type polycarbonate (PC) and a high permittivity poly(vinylidenefluoride-co-hexafluoropropylene) (PVDF-HFP), to assess the effect of polymer host dielectric permittivity on the dielectric properties of nanocomposites. PC has been extensively used for high-energy density capacitors because of its high dielectric strength and low dissipation factor. PVDF-HFP is a highly processable copolymer that is expected to have a high dielectric strength comparable to PVDF. Figure 4 illustrates the structures of the phosphonic acids used by Kim to create their polymer composites. 

**Figure 5:**
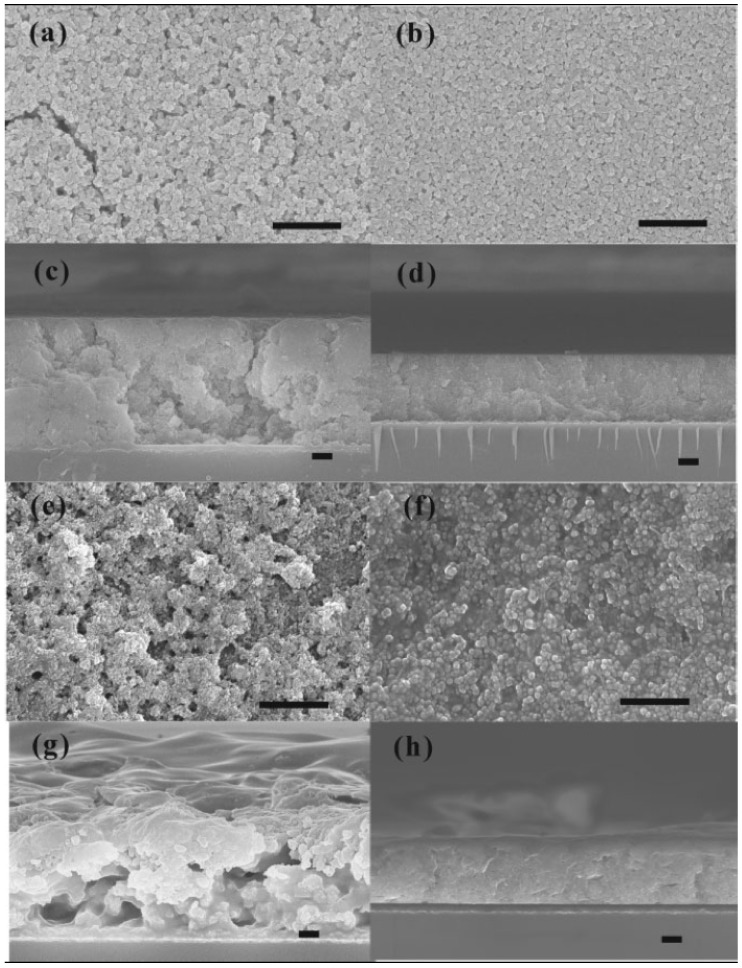
Surface and cross-sectional SEM images of spin-coated nanocomposite thin films. a,c) BT/PC, b,d) PEGPA-BT/PC, e,g) BT/PVDF-HFP, and f,h) PFBA-BT/PVDF-HFP. Figure reproduced from Kim *et al*. [38] with permission from Wiley-VCH Verlag GmbH & Co. KGaA.

The chemical modification of BT effectively decreased particle agglomeration in suitable organic solvents, which is a very important step in polymer-composite thin film preparation. Typically, the surface modification is more designed for the anticipatory organic solvent that will be used to dissolve the polymer than for the polymer itself. In a normal process, functionalized-BT particles are dispersed in an organic solvent that already contains dissolved polymer. The solutions are typically sonicated, and thin-films are produced by spin-coating. The surface and cross-section of the film are typically investigated by SEM to determine the uniformity of the nanocomposites. Figure 5 shows the surface and cross-sectional SEM images of Kim’s nanocomposites.

**Figure 6:**
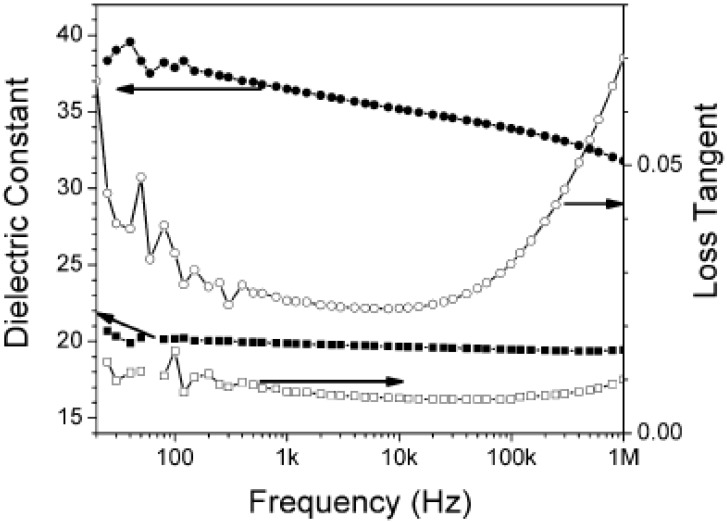
Frequency-dependent dielectric response of capacitor devices fabricated from PEGBA-BT/PC (squares) and PFBPA-BT/PVDF-HFP (circles). Figure reproduced from Kim *et al*. [38] with permission from Wiley-VCH Verlag GmbH & Co. KGaA.

The dielectric characterization and spectroscopic results of phosphonic acid-modified BT/ nanocomposites are summarized in Table 4 and Figure 6. A large dielectric permittivity and a reasonably low loss up to frequencies of 1 MHz were observed for both types of composites. The role of the polymer host dielectric permittivity is evident in the nearly two-fold increase of the effective dielectric permittivity observed for the PVDF-HFP compared to the PC composites. 

One of the most important attributes of a DPN is its maximum energy density. In part because of their large dielectric strength and high permittivity, as well as because of filler dispersion, Kim’s modified BT nanocomposites afford large calculated energy densities of up to 6.1 J cm^-3^. 

**Figure 7:**
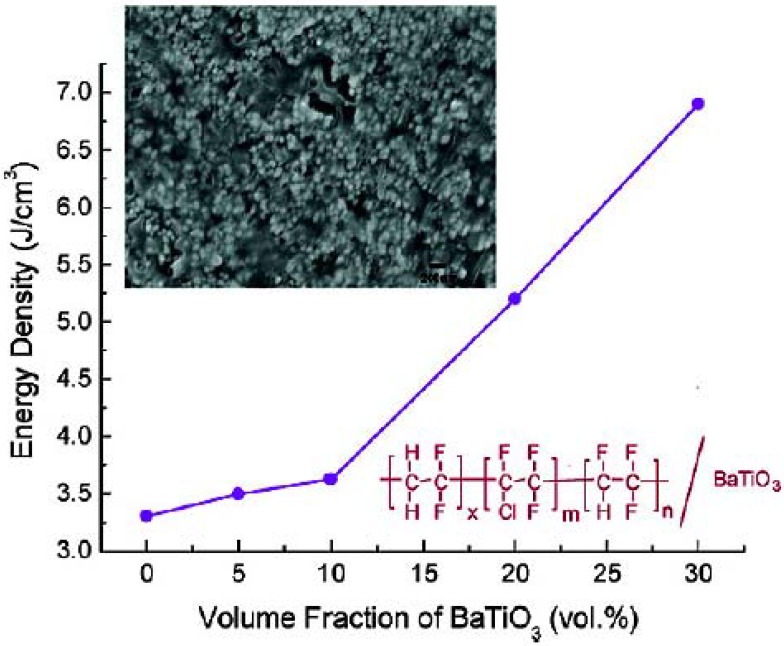
Cross-sectional FE-SEM image of PVDF-CTFE-BT nanocomposite thin film containing 23 vol % ethylenediamine-BT. Figure reprinted with permission from Wang *et al*. [39] Copyright 2008 American Chemical Society.

Wang’s work on modified-BT is similarly based on PVDF based polymers. The unique dielectric properties of PVDF based polymers originate from the presence of the highly electronegative fluorine on the polymer chains and from the spontaneous alignment of the C-F dipoles in the crystalline phases [58]. Again, the important aspect in DPN synthesis is the dispersion of inorganic fillers. In fluorinated polymers dispersion of BT is problematic due to the extremely low surface energy of the polymers. The agglomeration of ceramic dopants can give rise to electronic conduction that can lead to a high dielectric loss as well as to undesirable porosity and associated dielectric failure at low applied fields. Wang’s functionalization differs from Kim’s, in that Wang successfully produces nano-sized, ethylene diamine surface modified-BT *in situ*. As in the case of Kim *et al*., the surface modification afforded an enhancement in the dispersion into organic solvents and subsequently led to an improvement in the homogeneity of the resulting polymer films. Figure 7 inset shows the uniformity of the nanocomposite films through TEM and cross-sectional field-emission SEM. The dielectric permittivity steadily increases with the increase in modified-BT content. The electrical energy density of the nanocomposites as a function of filler concentration is also summarized in Figure 7. Clearly, the addition of modified-BT nanoparticles into polymers greatly increases the energy density of the composite materials. It is worth noting that at 150 MV/m, the stored energy density of the (PVDF-CFTE / 23 vol% modified-BT) composite is nearly doubled from 1.9 J cm^-3^ (pure PVDF-CFTE) to 3.7 J cm^-3^ for the composite.

**Table 4:** Dielectric characteristics of BT-nanocomposites. Table reproduced from Kim *et al*. [38] with permission from Wiley-VCH Verlag GmbH & Co. KGaA.

	50 vol % PEGBA-BT in PC	50 vol % PFBPA-BT in PVDF-HFP
Film Thickness (μm)	3.89	3.84
Capacitance density (nF/cm)	4.6 ± 0.6	8.6 ± 0.4
Relative permittivity at 1kHz	20 ± 2	37 ± 2
Dielectric Loss at 1MHz	<0.01	<0.07
Leakage current density (nA/cm^2^)	30	60
Dielectric Strength (V/μm)	210 ± 20	210 ± 50
Max energy density (J/cm^3^)	3.9	6.1

### 2.2. Titania (TiO_2_, TO) and TO Polymer Composites

The oxide TiO_2_ exists in three crystalline modifications at atmospheric pressure, namely rutile, anatase, and brookite. The rutile structure consists of chains of trans edge-sharing TiO_6_ octahedra where the chains, in turn, are connected by sharing corners. By comparison, the structure of anatase can be viewed as a framework of distorted TiO_6_ octahedra sharing four edges. This structural difference is believed to afford the rutile structure a higher dielectric permittivity than anatase.

As mentioned previously, it is thought that the inclusion of high dielectric permittivity fillers into relatively low dielectric permittivity polymers may not be ideal to achieve an appreciable increase in the energy density of the composite. This is due to the belief that if the filler has a much greater permittivity than the polymer matrix and because most of the increase in the effective dielectric permittivity comes through an increase in the average field in the polymer matrix, then very little of the energy will be stored in the high permittivity filler phase [59]. In addition, the presence of a large contrast in permittivity between the two phases generates a highly inhomogeneous electric field that significantly reduces the effective breakdown strength of the composite. For these reasons, some research groups focus on titania (TiO_2_, TO), since its average dielectric permittivity of ~47 is much closer to the typical permittivities of the polymer host material [60]. 

**Figure 8:**
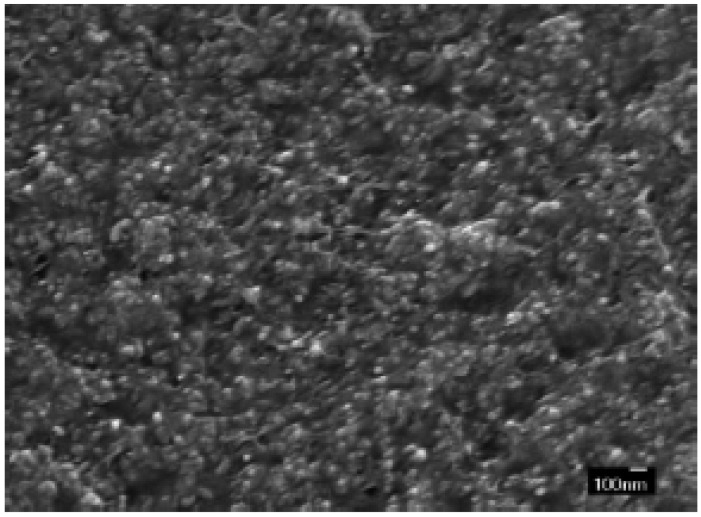
Cross-sectional FE-SEM image of 30 vol % TiO_2_/PVDF-TrFE-CTFE nanocomposite thin film. Figure reproduced from Wang *et al*. [60] with permission from Wiley-VCH Verlag GmbH & Co. KGaA.

For TO, also, surface functionalization is needed to prevent particle agglomeration within the polymer matrix by enhancing the particle-polymer matrix interaction. Wang’s group has shown excellent progress in the surface functionalization of TO for incorporation into the ferroelectric copolymer PVDF-TrFE-CTFE [60]. In this work, they prepared rod-shaped TO nanoparticles (~20 nm × 70 nm) via a hydrothermal reaction of titanium(IV)tetraisopropoxide and hydrogen peroxide. The hydrogen peroxide enables them to later functionalize or “coat” the surface of the rods with Ba-OH surface groups through a simple reflux reaction of the TO nanorods with CO_2_-free barium hydroxide solution under an Ar atmosphere. The Ba-OH surface groups exhibited good dispersion in DMF with an average aggregation size of ~60 nm and an overall size below 100 nm, as evidenced by DLS. Figure 8 shows the cross sectional FE-SEM image of the resulting 30 vol % modified-TO in PVDF-TrFE-CTFE.

**Figure 9:**
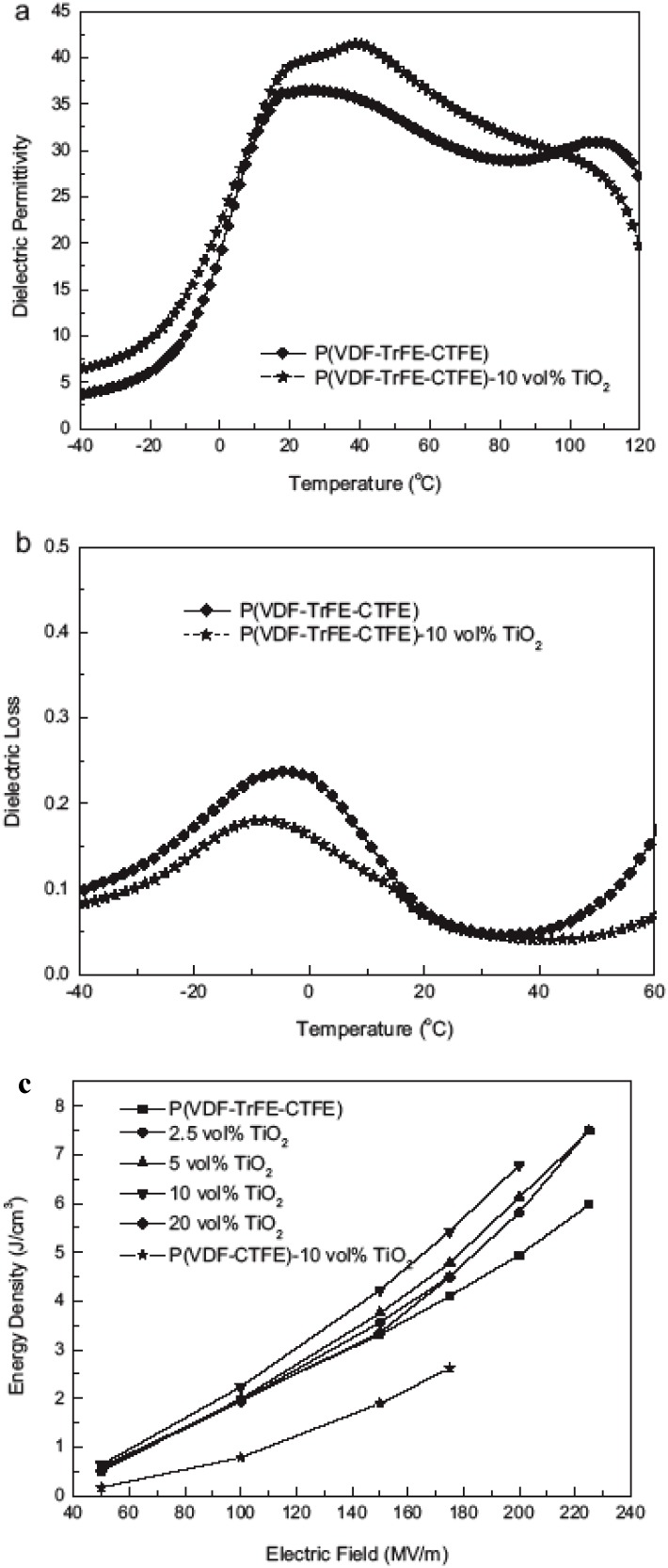
(a) Temperature-dependence of the dielectric constant of the polymer and nanocomposite measured at 1 kHz. (b) Temperature-dependence of the dielectric loss of the polymer and nanocomposite measured at 1 kHz. (c) The stored energy density of the polymer and nancomposites as a function of the applied field. Figures reproduced from Wang *et al*. [60] with permission from Wiley-VCH Verlag GmbH & Co. KGaA.

Figure 9a shows the gradual increase of the dielectric permittivity of TiO_2_/PVDF-TrFE-CTFE as a function of modified TO loading. Presumably because the permittivities of the two phases are similar, the dielectric loss, Figure 9b, shows very little variation with TO loading. The small variation in the dielectric loss is consistent with a well-dispersed and homogeneous TO-polymer composite. 

Figure 9c shows the stored electrical energy density of the TiO_2_/PVDF-TrFE-CTFE nanocomposites. The nanocomposites exhibit a much-improved energy density compared with the neat polymer. For 10 vol % modified-TO, the energy density is 6.9 J cm^-3^ at 200 MV m^-1^, which represents a ~45 % increase relative to the neat polymer matrix with an energy density of 4.7 J cm^-3^ at that same field. Figure 9c also compares the energy density of PVDF-TrFE-CTFE and PVDF-CTFE composites containing 10 vol % modified-TO nanoparticles. The fact that the PVDF-TrFE-CTFE composite has a higher energy density than the PVDF-CTFE composite is presumably due to the higher intrinsic permittivity of PVDF-TrFE-CTFE vs. PVDF-CTFE. 

It is interesting to note that the maximum energy density is obtained for 10 vol % modified-TO. Any further increase in filler loading results in a decrease in the energy density. This result is likely caused by effects related to the interfacial area in the composites. For a given particle size, a maximum interfacial area can be achieved beyond which additional filler cannot generate additional interfacial area, but instead can generate interfacial polarization that will effect the dielectric response of the composite [35,61,62].

Perhaps the best work on examining the interfacial effects of DPNs comes from Ma *et al*., who studied the influence of surface modification of TO nanoparticles on the short-term breakdown strength and space charge distribution of LDPE(low density polyethylene) [35]. They chose a polar silane coupling agent, AEAPS (*N*-(2-aminoethyl)-3-aminopropyl-trimethoxysilane), for the nanoparticle surface modification. Despite agglomeration and a poor interface compared to unmodified nanoparticles, they did find that the incorporation of polar groups onto the nanoparticle surface improved both the dielectric breakdown and space charge distribution as compared to the composites made from unmodified TO. They investigated three different polymer-composite systems: The first composite sample was neat LDPE (no filler) and was used as a control. The other two composites systems were comprised of as received TO and the other of AEAPS-TO. The TO-LDPE composite films were prepared via melt blending – heating the polymer to a molten state and blending in the filler using a Haake mixer. The effect of the surface modification on dispersion was analyzed by FE-SEM. While the dispersion was not excellent, the distribution of the particles was good. The polar groups of AEAPS created agglomerates on the order of 30 microns for AEPAS-TO apparently due to the hydrophilicity of the polar groups trying to be incorporated into organophilic LDPE. 

Particles can act as electrical defect centers in filled polymers and can distort and enhance the local electrical field, which can lead to a decrease in the dielectric breakdown strength relative to the polymer itself [32,63,64]. Several factors in addition to the difference in permittivity or conductivity between the particle and polymer influence the extent of the field distortion and include the particle size and the adhesion between polymer and particle. It turns out that larger particles create a larger local field distortion. Thus, if dispersion is the dominant factor, the AEAPS-TO, with large agglomerates should have had a lower breakdown strength than TO. The fact that AEAPS-TO showed a marked improvement in the dielectric breakdown strength suggests, however, that this is not a dominant effect here. Poor adhesion between the polymer and the particle also causes a reduction in the breakdown voltage [32]. The presence of polar groups on the surface of TO should create a more hydrophilic surface in the AEAPS-TO composite as compared to the as received TO and, thus, there should be decreased adhesion in AEAPS-TO with the hydrophobic LDPE. The fact that there is an increase in the breakdown strength in the AEAPS-TO/LDPE composite suggests that polymer-particle adhesion is not the dominant effect here. For these reasons, Ma *et al*. conclude that the polar groups act as charge scatters by trapping the electrons, thus decreasing the acceleration of electrons and therefore decreasing the cascading process leading to the initiation of dielectric breakdown. 

In a similar work, Tuncer *et al*. have incorporated nano-sized TO in polyvinyl alcohol (PVA) for cryogenic grid applications [65,66]. They observed an approximately 25 % increase in breakdown strength of the nano-TO filled PVA. However unlike the previous authors, they observed uniform distribution of TO particles. They attribute the consistent breakdown strength of the polymer embedded with fillers to this uniformity in filler dispersion.

### 2.3. CaCu_3_Ti_4_O_12_ (CCTO) and CCTO Polymer Composites

Miniaturization of electronic devices such as capacitors requires new dielectric materials with very high dielectric constant, which is generally obtained from ferroelectric and relaxor ferroelectric-based perovskites such as the previously described BT [67,68]. The discovery of giant dielectric (non-ferroelectric) materials, such as calcium copper titanate [69,70,71,72,73,74,75] and lanthanum strontium nickelate, discussed in the next section, have provided researchers with new materials for use in PDCs. 

**Figure 10:**
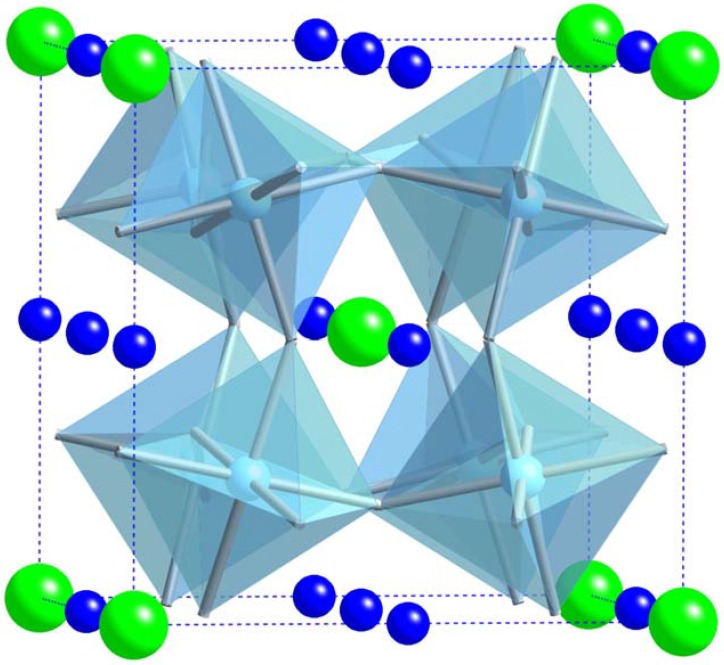
The unit cell structure of CCTO, with calcium ions in green, copper ions in blue, and TiO_6_ octahedra in teal.

**Figure 11:**
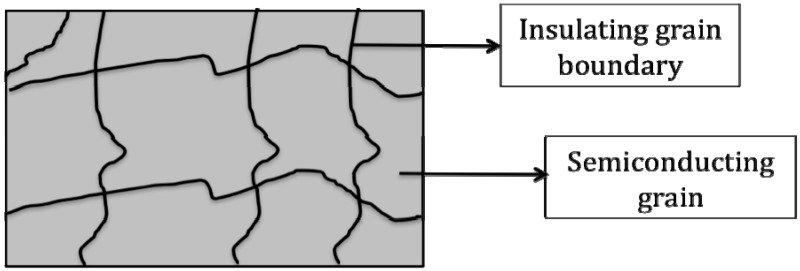
General schematic of the IBLC theory associated with CCTO’s giant dielectric constant. Schematic reproduced from Dang, Z.H. *et al*. [81] with permission from Wiley-VCH Verlag GmbH & Co. KGaA.

Recently, calcium copper titanate (CCTO), a perovskite-like (*ABO_3_*) body-centered cubic oxide, Figure 10, has attracted great attention for its giant dielectric constant. In CCTO, Ca and Cu ions reside at the *A*-sites, while Ti cations occupy the *B*-site. CCTO shows great promise in dielectric applications owing to its essentially temperature and frequency independent [76] dielectric constants ranging from about 10,000 for polycrystalline powders [77,78] to 100,000 for single crystals [70]. These excellent properties make CCTO a very attractive oxide for device application including PDCs [68,79]. To date, the origin of the giant dielectric constant in CCTO is still being debated. Homes *et al*. reported that below 100 K, the rapidly decreasing dielectric permittivity is due to ionic bonding in the TiO_6_ octahedra [70]. However, in the range of 100 K to 600 K, the origin of the high dielectric permittivity is argued to be due to both extrinsic and intrinsic factors, where the most widely accepted mechanism is the presence of an internal barrier layer capacitor (IBLC) [80,81]. The IBLC theory suggests that the giant dielectric permittivity arises from the oxidized insulating boundaries formed between the semiconducting grains. Figure 11 shows a schematic of IBLC theory with CCTO. It is however interesting, that the highest reported dielectric constants for CCTO have come from single crystal measurements, where there are no grain boundaries. This leaves open the possibility for another dielectric mechanism affording the high dielectric constant.

It appears that no one has reported a successful surface functionalization of CCTO to date, although several have tried. Consequently, some research groups have tried other approaches to facilitate the incorporation of CCTO into polymers. Dang *et al*. took as synthesized CCTO and incorporated it into polyimide (PI) through an *in situ* polymerization process. They report a dielectric permittivity value 14 times higher than neat PI in a 40 vol % CCTO-PI composite [81]. Prakash *et al*. added 25 vol % metallic aluminum powder into the CCTO-epoxy mixture, creating an Al-CCTO-epoxy three phase composite [82]. The three phase composite system has a dielectric permittivity of 700 at 300 K, which is a significant enhancement over the neat epoxy whose permittivity is 4.81.

Tuncer *et al*. have also investigated nano and sub-micron size CCTO fillers in epoxy resins [83]. They observe a slight reduction in dielectric permittivity of 5 % in CCTO composites compared to the base resin due to the loss of freedom for the matrix to relax under an external applied field. They also report a slightly lower breakdown strength for composites with heat-treated fillers, possibly due to aggregation of filler particles. However the composites had a higher reliability in dielectric breakdown strength, as indicated by the small difference between the maximum and minimum breakdown strengths observed. We can expect to see additional work on CCTO based DPNs in the future.

As mentioned earlier, much of the work in polymer dielectric nanocomposites are based on compromise. Thus the use of giant dielectric constant particles, such as CCTO, appears to result in dielectric loss in the composite systems. 

### 2.4. La_2-x_Sr_x_NiO_4_ (LSNO) and LSNO Polymer Composites

**Figure 12:**
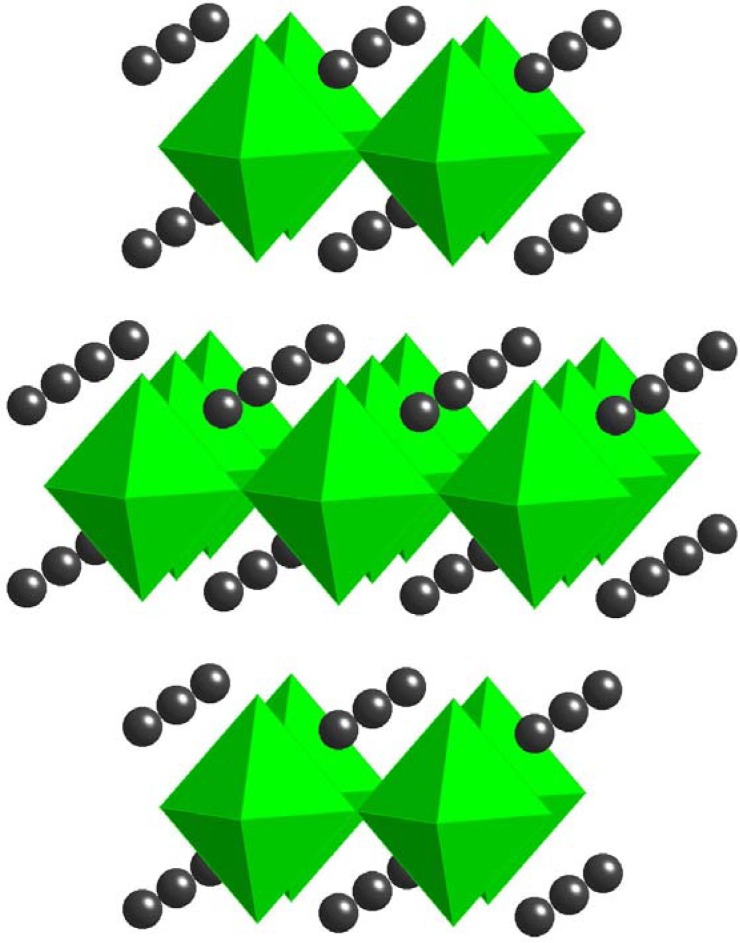
Tetragonal *K_2_NiF_4_*-type structure of La_2-x_Sr_x_NiO_4_. La ions are shown in black, Sr substitutes onto the La-site, and NiO_6_ octahedra are shown in green.

Another giant dielectric material that also may find application in the miniaturization of electronic devices, such as capacitors, is La_2-x_Sr_x_NiO_4_. It belongs to a family of compounds, Ln_2-x_Sr_x_NiO_4_ (Ln = La, Nd, Sm; x = 0.2 or 0.5), that show high dielectric permittivity (~10^5^) in the 5 MHz range over a broad temperature interval (150–500 K) [84] and are even stable (ε’ above 10^4^) at 1 GHz at room temperature [85]. These compounds form in the tetragonal K_2_NiF_4_ structure [86], shown in Figure 12.

**Figure 13:**
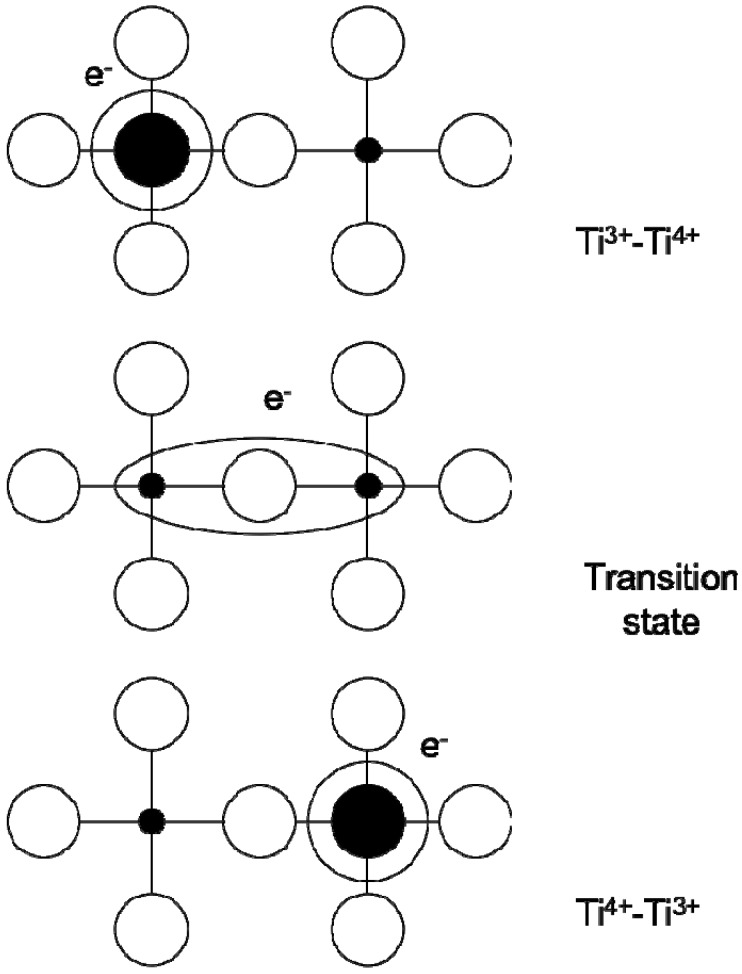
Schematic diagram of polaron electron transfer. Schematic reprinted with permission from Dupuis *et al*. [87] Copyright 2007 by the American Physical Society.

The observed giant dielectric constant is thought to be due to charge ordering in these structures, which leads to thermally activated small polaronic hopping. Electrical conduction in transition metal oxides occurs through strong coupling between electrons and phonons (from lattice vibrations), which create so-called *polarons*. The polarons can jump from one site (leaving a hole behind) to another (filling a hole) at the temperature greater than half of the Debye temperature (the temperature where charge ordering is observed in the structure) [87]. In the case of Ti-based giant dielectric constant materials such as reduced BT, the electrons from Ti^3+^ can jump into adjacent Ti^4+^ site, which involves antisymmetric displacement of bridging oxygen atoms as shown in Figure 13. 

This antisymmetric displacement of bridging oxygens causes a large overall polarization in the whole structure, which in turn leads to the giant dielectric constant. This thermally activated small polaronic hopping can be applied to other giant dielectric materials possessing holes in their structures, such as LSNO. In the LSNO compounds (*La_1.8_Sr_0.2_NiO_4_ and La_1.5_Sr_0.5_NiO_4_*), the thermally activated polaronic hopping occurs between the *Ni^2+^* and *Ni^3+^* metal ions at a temperature range between 200 K and 480 K [88].

Unfortunately, such a very high dielectric constant value is accompanied by a high dielectric loss due to the electron hopping. The electron hopping causes electrical conduction in these materials. Preventing the small polaronic hopping occurring across the grains of these oxides can dampen such high dielectric losses. One of the ways of accomplishing this is by encapsulating these oxides in a thin insulating shell. This method is commonly referred to as core-shell method, which will be discussed further in the next section. To date, no reports in the literature have been found on LSNO being functionalized or incorporated into polymers. 

## 3. Core Shell Nanoparticles and Percolating Inter-Particle Barrier Layering

Another approach researchers have studied in an effort to raise the energy density of polymer-composites is called core shell formation. In order to understand the usefulness of core shell synthesis and how it applies to polymer dielectrics, it is important to discuss the theory behind their application in dielectrics. Core shell nanoparticles belong to a class of dielectric materials known as percolative composite capacitors. In these composites, highly conductive fillers (e.g., metal particles) can lead to conductivity it they are present at the proper concentration [52,89,90]. As the volume fraction *f* of the fillers increases to the vicinity of the percolation threshold *f_c_*, κ of the composites can be dramatically enhanced as described by well-known power law [91,92]:

κ/κ_m_ ≈ | *f_c_ – f* |*^-s^*

where κ_m_ is the dielectric constant of the matrix and *s* is an exponent of about 1. Dielectric constant enhancement, κ/κ_m_, of about 10 to 100 has been observed in a number of such percolative polymer composites [52,89,90,93,94,95]. However, simultaneously, the dielectric loss of these percolative composites increases rapidly (e.g., from 2% for the polymer matrix to over 20%) due to the insulator-conductor transition near *f_c_* [52,89,90]. Therefore attempts to reduce the dielectric loss have been made by introducing interlayers or shells between the metal fillers to prevent them from connecting with each other directly [54,95]. Although the dielectric loss of these metal-polymer composites with surface-modified or surface-oxidized metal particles remained rather low (~5%), a serious variation in the dielectric constant (κ) was still observed when *f* is very close to *f_c_*. 

**Figure 14:**
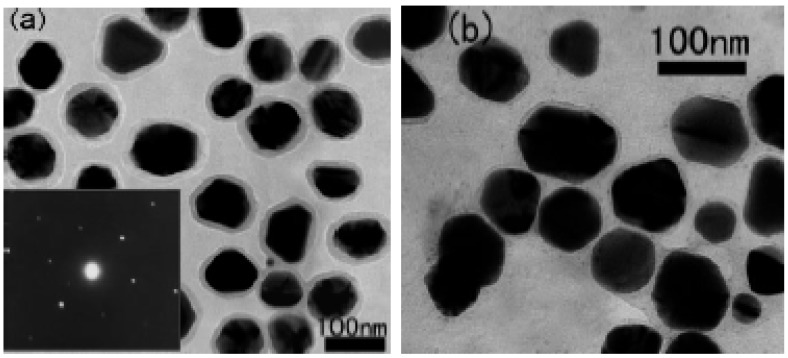
TEM image of silver-organic shell particles with a) thicker shells and b) thinner shells. The inset is the electron diffraction pattern of the particles, and illustrates the crystallinity of the silver cores. Images reproduced from Shen *et al*. [31] with permission from Wiley-VCH Verlag GmbH & Co. KGaA.

**Figure 15:**
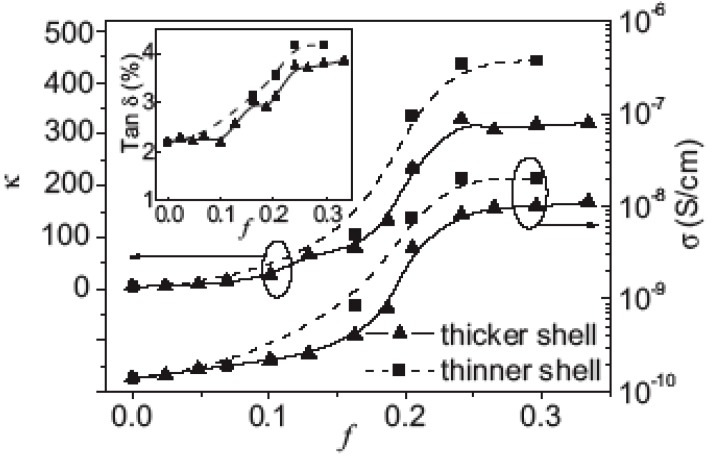
Variations of dielectric constant (κ), conductivity (σ), and dielectric loss (tan δ) of the composite films containing the silver-organic shell with thicker shells or thinner shells measured at 1 kHz. Plots reproduced from Shen *et al*. [31] with permission from Wiley-VCH Verlag GmbH & Co. KGaA.

Nan *et al*. reported an extremely stable high-κ with a low dielectric loss polymer-composite dielectric by coating silver metal particles with organic dielectric shells [31]. The metal-core shell particles, which consisted of glucose and poly(vinyl pyrrolidone) were hydrothermally treated with silver nitrate. The organic dielectric shells not only act as interparticle barriers to prevent direct connection of silver particles, but they also produce excellent compatibility between the filler and polymer to ensure the dispersion of the fillers within the polymer matrix. Figure 14 shows TEM images of the silver-organic shell particles, which average 80 nm in diameter. The organic shells average 8 to 10 nm in thickness for the thicker shells and 4 to 6 nm for the thinner shells. Figure 15 shows that adding the silver-organic shell fillers to the epoxy increases the permittivity by two orders of magnitude.

**Figure 16:**
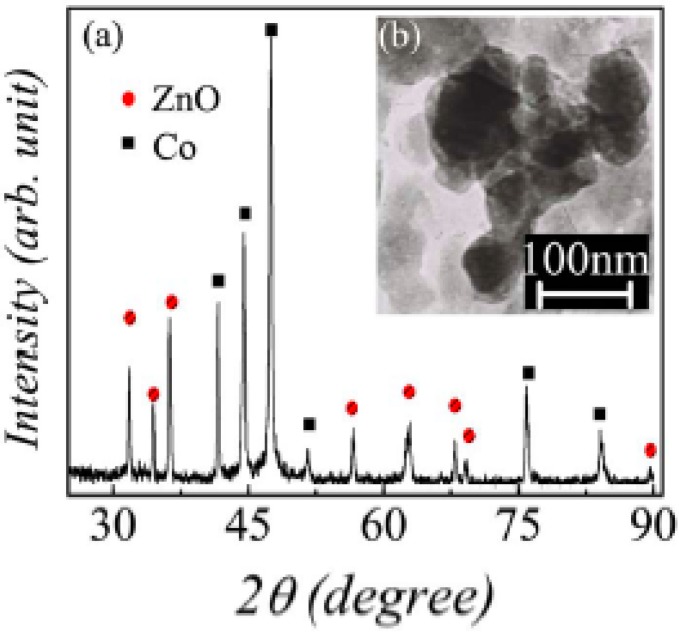
(a) XRD pattern and (b) TEM image of the as prepared Co/ZnO core/shell nanoparticles. Images reprinted with permission from Liu *et al*. [94] Copyright 2007, American Institute of Physics.

Another example of core shell nanoparticles comes from Liu *et al*., where cobalt particles were coated with a zinc oxide shell, which was further surface modified with organophosphorous acid [94]. The Co/ZnO core shell particles were incorporated in PVDF. The Co/ZnO particles measured 100 nm for an average diameter with 15 nm shell thicknesses, which is shown in Figure 16. 

**Figure 17:**
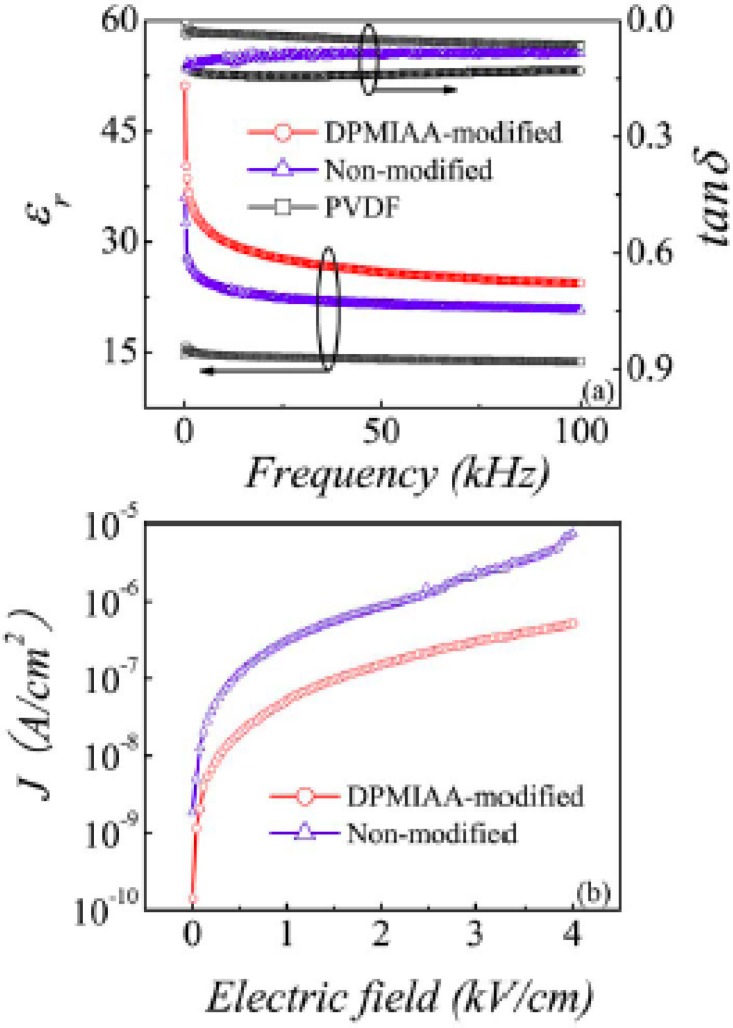
(a) Variation of dielectric constant with frequency for pure PVDF, PVDF composites of unmodified-Co/ZnO and DPMIAA-modified Co/ZnO particles. (b) DC leakage current of PVDF-unmodified Co/ZnO and PVDF-DPMIAA-modified-Co/ZnO composites. Plots reprinted with permission from Liu *et al*. [94] Copyright 2007, American Institute of Physics.

Figure 17 shows the variation of the dielectric permittivity and the loss factor with frequency for the three samples: pure PVDF, un-modified Co/ZnO-PVDF composite, and modified Co/ZnO-PVDF composite. It is apparent that the permittivity decreases for all the samples as the frequency increases. While the unmodified Co/ZnO-PVDF composite has twice the permittivity over that of pure PVDF, the permittivity value of the modified Co/ZnO-PVDF composite is three times higher than that of pure PVDF. Both the modified and unmodified Co/ZnO-PVDF have a maximum dielectric loss of ~0.14, much lower than conductive filler composites and polymer-ceramic composites. One interesting point is that the dielectric loss was slightly higher for the modified core shell than for the unmodified filler. This could be explained by the phosphonic ligands responding to the external ac signal. In terms of the leakage current, which can also be seen in Figure 17, the modified sample has a much lower leakage current than the unmodified one. 

The reasons responsible for the improved electrical properties are believed to be as follows. First, the reduced leakage current is attributed to the surface modification by DPMIAA, shown in Figure 16, which provides an efficient surface passivation that depresses the mobility of charge carriers associated with the surface of the Co/ZnO additive. Secondly, the concentration of ionizable hydroxyl groups on the nanoparticle surface is minimized due to the passivation layers [38]. Thirdly, the ZnO shell may also function as a barrier layer against conduction by preventing direct contact of Co particles, although ZnO is a semiconductor. The high dielectric permittivity of the modified and unmodified samples is evidently ascribed to the fact that the conducting particles are isolated by the thin dielectric layers to form microcapcitors, thus preferring the enhancement of the effective capacitance. 

## 4. Non-Oxide Dielectric Materials: Mixed-Metal Phenyl Phosphonates

New layered 1:1 mixed Ba^2+^/Ti^4+^ metal phosphonates, BaTi(C_6_H_5_PO_3_)_3_ and SrTi(C_6_H_5_PO_3_)_3_, have been prepared from reacting BaTiO_3_ and SrTiO_3_ with phenyl phosphonic acid [96]. The mixed-metal phosphonates were combined with polystyrene (PS) via a solution route and spin casted into thin films. The reaction between ternary oxides, BaTiO_3_ and SrTiO_3_, and phenyl phosphonic acid is similar to the well-known dissolution reactions between mixed metal oxides and chelating species, such as oxalic acid, which result in mixed metal oxalates. Moreover, during such dissolution reactions it has been determined that, after an initial incongruent dissolution of a small fraction of the solid, the dissolution becomes congruent and both metals are transferred at the same rate. In the case of tridentate phenyl phosphonic acid moiety, the resultant product is not a molecular metal complex but rather a mixed-metal extended structure that precipitates out of solution.

**Figure 18:**
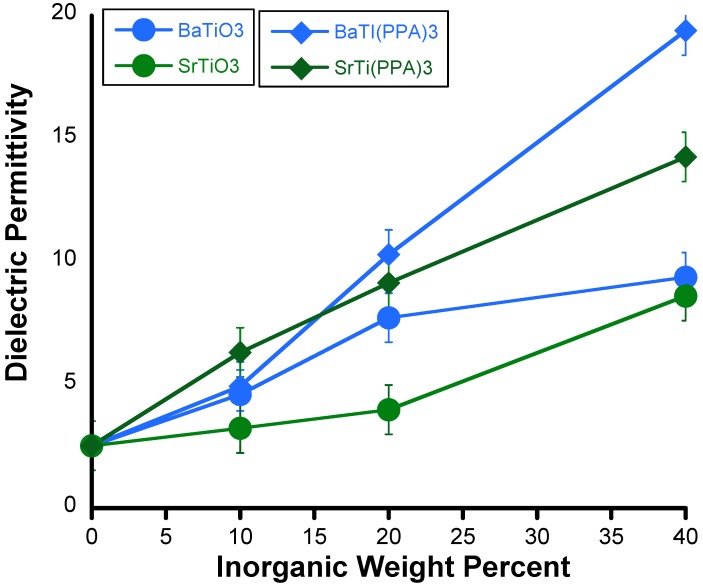
Dielectric permittivity values of polystyrene composites as a function of weight loading. Reprinted with permission from Barber *et al*. [92] Copyright 2009, American Chemical Society.

Polystyrene films have a reported dielectric permittivity of ~2-3, as shown earlier in Table 2. The addition of the mixed-metal phenyl phosphonates greatly increased the dielectric constant of the composite to up to ~20 for a 40 wt % loading of BaTi(C_6_H_5_PO_3_)_3_ in PS, as shown in Figure 18. The corresponding strontium analogue in PS reached a dielectric constant of ~14 as also shown in Figure 18. By comparison, the dielectric constants of the composites made by dispersing BaTiO_3_ or SrTiO_3_ in PS reached values of only ~9 in both cases. Clearly, the addition of the mixed-metal phosphonates stacks to the polymer resulted in an almost 10-fold improvement in the dielectric constant of the composite films. It is also important to point out that composites films prepared from single metal phosphonates, Ti(C_6_H_5_PO_3_)_2_ and Ba(C_6_H_5_PO_3_)●H_2_O, did not generate any improvement over the dielectric constants measured for BaTiO_3_ or SrTiO_3_ containing samples. Hence it would appear that either the presence of both metals or the structural difference between single and mixed-metal phosphonates leads to a significant enhancement of the dielectric constant.

## 5. Conclusions

In summary, this paper has reviewed some of the recent literature in the area of polymer composite and nanocomposite dielectric materials for energy storage and pulse power applications. The primary focus has been on inorganic filler materials and their polymer composites. The goal of most research efforts has been to incorporate inorganic fillers with large dielectric constants into polymer matrices with high breakdown field strengths, in the hopes of synthesizing composites with the best attributes of each component. A wide variety of inorganic fillers have been used, ranging from ones with relatively low dielectric constants, like TiO_2_, to materials with “giant” dielectric constants such as CaCu_3_Ti_4_O_12_. In most cases, the results are somewhat disappointing: the composites’ effective dielectric constants are much less than would be expected from the weight or volume average of the components’ dielectric constants. Moreover, the addition of inorganic filler tends to decrease the breakdown field strength to values considerably less than those of the bulk polymer.

One trend clearly emerges from recent work: for both many polymer composites and nanocomposites, the polymer-filler interface plays a critical role. Several studies show that appropriate surface modification of the inorganic filler can enhance the compatibility of the filler with the polymer. This has several consequences. First, favorable polymer-filler interactions promote better dispersion of the filler in the polymers, and this is empirically associated with higher effective dielectric constants of the composites. Second, better polymer-filler compatibility can improve the stability and specific surface area of the polymer-filler interface, possibly minimizing defects or voids in the composite that can decrease the breakdown strength and thus the overall energy density. On the other hand, surface modification of the filler particles introduces another component that can have unintended consequences, including higher ionic conductivity and dielectric loss. These undesirable side effects are amplified as the polymer-filler interfacial area increases. 

**Figure 19:**
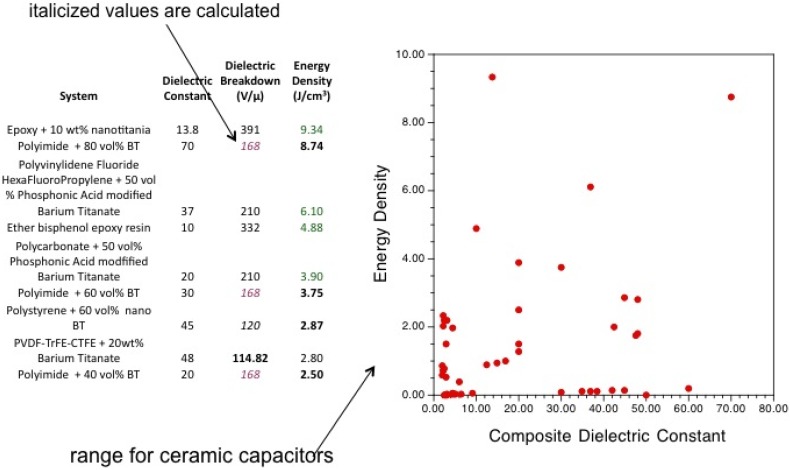
A compilation of reported and calculated energy densities from the literature for polymer-ceramic composites.

From the work reported in this review, we conclude that significant strides have been made in the development of polymer composites and nanocomposites with greater energy storage capacity and higher energy density, as shown in Figure 19. Still, much more work needs to be done to begin designing polymer composite dielectrics from first principles, rather than by mixing components in the hopes of finding improved properties. For the foreseeable future, the leading edge of research in this area will continue to focus on better understanding the chemistry and structure of the polymer-filler interface and its influence on composite dielectric permittivity and breakdown field strength.
